# Change in Substance Use and the Effects of Social Distancing on Health-Related Quality of Life and Depressive Symptoms During the COVID-19 Pandemic in People Living With and Without HIV

**DOI:** 10.1097/QAI.0000000000003055

**Published:** 2022-07-13

**Authors:** Reja E. A. Schaaf, Myrthe L. Verburgh, Anders Boyd, Ferdinand W. Wit, Pythia T. Nieuwkerk, Maarten F. Schim van der Loeff, Peter Reiss

**Affiliations:** a Department of Infectious Diseases, Amsterdam University Medical Centers, Amsterdam Institute for Infection and Immunity, Amsterdam, the Netherlands;; b Department of Global Health, Amsterdam Institute for Global Health and Development, Amsterdam, the Netherlands;; c HIV Monitoring Foundation, Amsterdam, the Netherlands;; d Department of Infectious Diseases, Public Health Service of Amsterdam, Amsterdam, the Netherlands; and; e Department of Medical Psychology, Amsterdam University Medical Center, University of Amsterdam, Amsterdam, the Netherlands.

**Keywords:** social distancing, health-related quality of life, depressive symptoms, substance use, HIV, COVID-19

## Abstract

Little is known about the impact of social distancing on health-related quality of life and depressive symptoms in older people with HIV during the COVID-19 pandemic.

**Setting:**

HIV-positive and HIV-negative AGE_h_IV Cohort Study participants.

**Method:**

In September-November 2020, participants completed questionnaires on social distancing, change in substance use, health-related quality of life (EQ-6D, including EQ-VAS), and depressive symptoms (PHQ-9). Associations between social distancing and (1) EQ-VAS or (2) PHQ-9 score ≥10 (clinically relevant depressive symptoms) were analyzed using fractional and binomial logistic regression, respectively.

**Results:**

Two hundred fourteen HIV-positive and 285 HIV-negative participants were analyzed. 77.4% found social distancing important and 66.9% reported good adherence to these measures, without significant differences between HIV-positive and HIV-negative participants. In both groups, <5% reported increased smoking or recreational drug use, but more HIV-positive (12.2%) than HIV-negative (4.9%) participants (*P* = 0.005) reported increased/more frequent alcohol use. Median EQ-VAS was slightly lower in HIV-positive (80 IQR = 73–90) than HIV-negative (84 IQR = 75–90) participants (*P* = 0.041). The prevalence of clinically relevant depressive symptoms was similar (HIV-positive, 8.4% and HIV-negative, 8.8%). Worrying about contracting COVID-19 and having ≥3 (vs no) comorbidities were associated with lower EQ-VAS and finding social distancing easy with higher EQ-VAS. Worrying about contracting COVID-19 and younger than 60 years (vs ≥65) were associated with higher odds of clinically relevant depressive symptoms. HIV status was associated with neither outcome.

**Conclusions:**

Initially during the COVID-19 pandemic in the Netherlands, a similar majority of HIV-positive and HIV-negative participants reported adhering to social distancing. Irrespective of HIV status, concerns about contracting COVID-19 negatively affected participants' perceived current health and increased risk of depressive symptoms.

## Abstract 2

Supplemental Digital Content is Available in the Text.

## INTRODUCTION

In January 2020, severe acute respiratory syndrome coronavirus 2 (SARS-CoV-2) was identified as the cause of coronavirus disease 2019 (COVID-19). In the Netherlands, the first case of COVID-19 was diagnosed on February 27, 2020.^1^ At the time, in the absence of widespread testing capacity, effective treatment, or vaccination, measures including social distancing, contact tracing, and quarantine were implemented in March 2020 as the mainstay public health measures to try and control the pandemic. Around May/June 2020, restrictions were eased: Public venues and indoor facilities reopened with face masks remaining mandatory in public transport. In anticipation of the “second wave” of SARS-CoV-2 infections, stricter measures were enforced again in September 2020, including working from home, mandatory use of face masks in indoor public venues, and closure of restaurants and bars from October until November 2020.^1^

Not much is known about the impact of social distancing measures in people living with HIV (PLWH). One longitudinal cohort study in 183 PLWH and 116 HIV-negative people in Miami, FL, found that 97.3% of the participants, of whom 5.6% had been tested positive for COVID-19, practiced social distancing, without a significant difference between HIV-positive and HIV-negative participants.^2^

One could expect that use of substances, such as tobacco, alcohol, and/or recreational drugs, might increase during the COVID-19 pandemic, because of imposed social distancing measures and related depression.^3,4^ One study found an increase in frequency, duration, and amount of tobacco use during the pandemic among PLWH and a similar increase in alcohol consumption among PLWH and HIV-negative participants.^5^ Another study found a significant decrease in the proportion of PLWH who smoked.^2^

Concerns have also been raised regarding the psychosocial impact of social distancing, in particular on quality of life (QoL) and depression. Among PLWH, currently no data are available on changes in health-related QoL (HRQoL) during the COVID-19 pandemic. Concerning depression, one US study, among 133 PLWH and 54 demographically similar people without HIV, found those with HIV to have a significantly higher Beck Depression Inventory II score. Both groups had a similar increase in this score compared with before the COVID-19 pandemic.^5^

Social distancing measures may continue to be required to a varying extent until the SARS-CoV-2 pandemic has truly been contained. Similarly, such measures may again be required in case of future emergence of respiratory pathogens with pandemic potential. Thus, it is important to learn from the current COVID-19 pandemic and gain insight into the possible psychosocial and behavioral effects of social distancing, including in PLWH.

The aim of this study was to compare change in substance use and the experience with and adherence to social distancing measures between HIV-positive and HIV-negative participants of the AGE_h_IV Cohort Study and to assess the impact of social distancing on self-reported HRQoL and depressive symptoms between both groups.

## METHODS

### Study Population

The AGE_h_IV Cohort Study is a prospective observational cohort study assessing the prevalence and incidence of age-related comorbidities and their risk factors in HIV-1–positive and HIV-negative participants aged 45 years or older at the moment of inclusion. Between 2010 and 2012, HIV-positive participants were recruited at the outpatient HIV clinic of the Amsterdam University Medical Centers (UMC), location Academic Medical Center (AMC), and HIV-negative participants from either the sexual health clinic or the Amsterdam Cohort Studies on HIV/AIDS at the Public Health Service Amsterdam, resulting in a control group with highly similar sociodemographic and behavioral characteristics. At baseline and every 2 years thereafter, participants undergo standardized screening for age-related comorbidities and collection of blood, urine, and stool for cryopreservation. Details have been described previously.^6^

In August 2020, after the first SARS-CoV-2 epidemic wave in the Netherlands, all AGE_h_IV Cohort Study participants in active follow-up and residing in the Netherlands were asked to participate in a COVID-19 substudy, which includes 5 planned, 6-monthly study visits between September 2020 and October 2022. During each visit, participants complete a standardized study questionnaire, and blood is obtained to assess SARS-CoV-2 humoral and cellular immune responses.^7^

Written informed consent was obtained from all participants. This study was approved by the Ethics Committee of the Amsterdam UMC, location AMC and was registered at www.clinicaltrials.gov (NCT01466582).

### Data Collection

Baseline characteristics, including date of birth, self-identified sex, ethnic origin, educational level, number of comorbidities, and lifestyle factors, such as smoking, alcohol use, and recreational drug use, were already available as part of the general AGE_h_IV Cohort Study data collection. An educational level was defined as the highest education achieved in 2010–2012 (baseline AGE_h_IV study visit). This was categorized as lower education, defined as primary and secondary education and lower vocational education; higher education, defined as higher vocational education and university; and others. In the COVID-19 substudy, additional substudy-specific data were collected using a 6-monthly administered questionnaire (Supplemental Digital Content 1, http://links.lww.com/QAI/B930).

For the current analysis, COVID-19 questionnaire data collected during the first substudy visit (September–November 2020) were used. Participants who indicated in the questionnaire that they had tested positive for SARS-CoV-2 or had been diagnosed with COVID-19 were excluded from the analysis, given that their experience with and adherence to social distancing, HRQoL, and reporting of depressive symptoms might have been different because of their infection.

### Study Variables

Baseline *age* (defined as age at the moment of completing the questionnaire) was categorized as younger than 60, 60–64, 65–69, and 70 years and older. The *number of comorbidities* as per the last available parent cohort study visit was categorized as 0, 1–2, and 3–7. *Experience with and adherence to social distancing* were assessed with questions about self-perceived adherence, the experienced difficulty with and importance of these measures, and number of household members and their adherence to social distancing according to the participant. Moreover, 2 questions explored the self-reported concern of getting infected with SARS-CoV-2 and the importance of preventing infection. All questions were answered on a 7-point Likert scale. The response to questions on experience with and adherence to social distancing was categorized to ensure comparisons with sufficient numbers of participants (see Table S1, Supplemental Digital Content 3, http://links.lww.com/QAI/B932). Self-reported *change in* s*moking*, *alcohol use*, *and drug use* were assessed with questions concerning the change in substance use since the start of the COVID-19 pandemic. The participants chose one of the following answers which best described their situation: “I use (…) more or more often,” “I use (…) as much as before/I never used it,” “I use (…) less or less often,” or “I stopped using (…),” whereby (…) in actual questions refers to tobacco, alcohol, or recreational drugs.

The EQ-6D questionnaire^8^ was used to assess participants' current *health-related quality of life*, scored on 6 domains (mobility, self-care, usual activities, pain, anxiety/depression, and cognition) and also includes a score from 0 (worst possible health) to 100 (best possible health), whereby participants score their current perceived health (EQ-VAS). The PHQ-9 scale was used for scoring *depressive symptoms*,^9,10^ with a PHQ-9 score ≥10 considered indicative of clinically relevant depressive symptoms, with a sensitivity and specificity for major depression of 88%.^10^ The PHQ-9 score was analyzed both as a continuous variable and dichotomized (at the before mentioned cut-off of ≥10).

### Statistical Analysis

Baseline characteristics of participants consenting to be included in the COVID-19 substudy and those that did not were compared using Pearson Χ^2^ or Mann–Whitney *U* test, as appropriate. Baseline characteristics, experience with and adherence to social distancing, number of household members, change in substance use, EQ-6D, EQ-VAS, and the PHQ-9 score were described using absolute numbers and percentages or medians and interquartile range (IQR), as appropriate. To compare these values between HIV-positive and HIV-negative participants, the Pearson Χ^2^ or Mann–Whitney *U* test was used, as appropriate.

The association between social distancing variables and 2 outcomes was examined: (1) HRQoL, as determined by the EQ-VAS, and (2) clinically relevant depressive symptoms. EQ-VAS levels were transformed to values between 0 and 1 and modeled using fractional logistic regression. Coefficients represent the change in back-transformed score compared with the reference of the independent variable and were estimated along with their 95% confidence intervals (CI) using this model. The presence of clinically relevant depressive symptoms was modeled using logistic regression. Odds ratios (OR) comparing the odds per unit increase of the independent variable and their 95% CI were estimated using this model. Age, self-identified sex, ethnic origin, HIV status, educational level, household size, number of comorbidities, and change in substance use were included in univariable analysis. All factors univariably associated with the outcome measure at a *P* < 0.20 were subsequently included in a multivariable model and removed in backward stepwise fashion when *P* ≥ 0.05, while HIV status was forced in the final multivariable model. Interactions between social distancing parameters and HIV status were tested in the multivariable model and included when *P* < 0.05.

To assess the relationship between social distancing patterns and (1) HRQoL and (2) clinically relevant depressive symptoms, a latent class analysis (LCA) was performed *post hoc*.^11^ Categorical values of “being worried about getting COVID-19,” “adherence to social distancing measures,” and “experienced difficulty with social distancing” were included in a LCA model. Models with increasingly higher numbers of latent classes were sequentially run, and the model with the lowest Bayesian Information Criteria score was selected. The a posteriori probability of belonging to each latent class was calculated for each participant, and class membership was assigned based on the highest class probability. Individual social distancing measures were replaced by the social distancing class in the 2 final multivariable models assessing associations between social distancing and (1) HRQoL and (2) clinically relevant depressive symptoms.

Individuals with missing data for a given analysis were excluded from that specific analysis. A *P* < 0.05 was defined as statistically significant. All statistical analyses were performed using STATA (version 15.1; College Station, TX).

## RESULTS

### Baseline Characteristics

In August 2020, all 824 AGE _h_IV Cohort Study participants in active follow-up were informed about the COVID-19 substudy, and 238 HIV-positive and 312 HIV-negative participants consented to participate. Of those, 218 HIV-positive and 294 HIV-negative participants completed the substudy questionnaire. Thirteen individuals (4 HIV-positive and 9 HIV-negative) were excluded from the current analysis because they reported to have tested positive for SARS-CoV-2 and/or had been diagnosed with COVID-19. This resulted in a total of 214 HIV-positive and 285 HIV-negative participants being included in the current analysis (Supplemental Digital Content 2, http://links.lww.com/QAI/B931). Compared with included participants, those who were excluded or declined participation were significantly more often HIV-positive, of African origin, and had attained lower education levels (see Table S2, Supplemental Digital Content 3, http://links.lww.com/QAI/B932).

Most of the participants were White and male. The median age was 60.9 (interquartile range (IQR) 57.2–66.7) years, and 55.9% of participants were highly educated. Compared with HIV-negative participants, HIV-positive participants were significantly older, more often male, and had more comorbidities (Table 1). Before the COVID-19 pandemic, a minority of participants currently smoked (20.4%) and/or used recreational drugs (34.0%), whereas most of them reported alcohol use (83.8%). Participants who reported using recreational drugs most frequently used cannabis (43.6%), 3,4-methylenedioxymethamphetamine (MDMA) (23.9%), and cocaine (19.0%). Significantly more HIV-negative than HIV-positive participants used alcohol; yet, there was no difference in the frequency of alcohol use between the groups.

**TABLE 1.** Characteristics of AGEhIV COVID-19 Substudy Participants (in Amsterdam From September 2020 Until November 2020) Included in the Analysis, by HIV Status

	HIV-Positive Participants (n = 214) No. (%) or Median (IQR)	HIV-Negative Participants (n = 285) No. (%) or Median (IQR)	*P*
Demographics			
Self-identified female sex	15 (7.0)	48 (16.8)	**0.001** *
Age, y			0.093*
<60	84 (39.3)	138 (48.4)	
60-64	52 (24.3)	65 (22.8)	
65-69	47 (22.0)	41 (14.4)	
≥70	31 (14.5)	41 (14.4)	
Ethnic origin			0.066*
White	206 (96.3)	269 (94.4)	
African	8 (3.7)	9 (3.2)	
Asian	0 (0.0)	7 (2.5)	
Educational level ^A,B^			0.536*
Lower education	96 (45.1)	115 (40.6)	
Higher education	113 (53.1)	164 (58.0)	
Others	4 (1.9)	4 (1.4)	
Number of comorbidities			**<0.001** *
0	94 (43.9)	175 (61.4)	
1–2	99 (46.3)	95 (33.3)	
3–7	21 (9.8)	15 (5.3)	
Behavioral characteristics ^C,D^			
Cigarette smoking	39 (18.2)	63 (22.1)	0.287*
Daily number of cigarettes smoked ^E^	10 (3–15)	6 (2–20)	0.860†
Alcohol use	170 (79.4)	248 (87.0)	**0.023** *
Frequency of alcohol use ^F^			0.114*
Less than monthly	10 (5.9)	21 (8.5)	
Monthly	22 (12.9)	40 (16.1)	
Weekly	63 (37.1)	106 (42.7)	
Daily or almost every day	75 (44.1)	81 (32.7)	
Recreational drugs use ^G^	67 (32.5)	96 (35.0)	0.565*

Missing data were not considered in the comparisons. Owing to rounding and missing data, percentages within variables might not add to exactly 100%. ^A^Data from the first study round of the AGE_h_IV Study;^B^ Missing data from 1 (0.47%) HIV-positive participant and 2 (0.70%) HIV-negative participants;^C^ Last available data before the start of the COVID-19 pandemic (February 2020);^D^ In the 6 months before answering the question;^E^ In those who smoke;^F^ In those who use alcohol;^G^ Missing data from 8 (3.7%) HIV-positive participants and 11 (3.9%) HIV-negative participants.

* Pearson χ^2^.

† Mann–Whitney *U* test.

Ref, Reference.

### Social Distancing

Most of the participants found it important to prevent getting infected with SARS-CoV-2 (79.8%), found social distancing measures important (77.4%), and reported both a good self-adherence (66.9%) and good adherence by their household members (62.9%) to these measures. Of all the participants, 30.1% were worried about getting ill with SARS-CoV-2 and 20.1% found social distancing difficult. No significant difference was found between HIV-positive and HIV-negative participants, regarding their self-perceived experiences with and adherence to social distancing (see Table S3, Supplemental Digital Content 3, http://links.lww.com/QAI/B932).

### Change in Substance use

Most participants reported no change in/never having used tobacco, alcohol, or drugs (85.9%, 73.2%, and 86.7%, respectively). A minority of participants reported increased or more frequent use of tobacco, alcohol, or recreational drugs (3.8%, 8.1%, and 2.4%, respectively). Compared with HIV-negative participants, significantly more HIV-positive participants reported increased or more frequent use of alcohol since the start of the COVID-19 pandemic (*P* = 0.005). There were no significant differences between HIV-positive and HIV-negative participants in self-reported change in smoking (*P* = 0.777) or recreational drugs use (*P* = 0.985) (see Table S4, Supplemental Digital Content 3, http://links.lww.com/QAI/B932).

### Self-Reported Health-Related Quality of Life

Most of the participants reported no problems in the domains of mobility (82.4%), self-care (95.8%), performance of usual activities (80.5%), pain (57.8%), anxiety/depression (61.9%), and cognition (72.1%), without significant differences between HIV-positive and HIV-negative participants, except for significantly more HIV-positive than HIV-negative participants reporting slight or moderate problems in the domain mobility (22.0% vs 11.2%, respectively (*P* = 0.011)) (see Table S5, Supplemental Digital Content 3, http://links.lww.com/QAI/B932). HIV-positive participants scored their perceived current health significantly, albeit only slightly lower, than HIV-negative participants [median EQ-VAS 80 (IQR 73–90) for HIV-positive vs 84 (IQR 75–90) for HIV-negative participants (*P* = 0.041)].

### Self-Reported Depressive Symptoms

The median PHQ-9 score was 3 (IQR 1–6) for HIV-positive participants and 3 (IQR 1–5) for HIV-negative participants (*P* = 0.889). Similar proportions of participants (8.4% of HIV-positive and 8.8% of HIV-negative participants (*P* = 0.887)) met the criteria for clinically relevant depressive symptoms (PHQ-9 score ≥10).

### Association Between Social Distancing and Current Perceived Health (EQ-VAS)

Being worried about getting ill with COVID-19 (difference −5.15 [95%CI −7.92 to −2.38]) and having ≥3 comorbidities (difference −10.02 [95%CI −15.55 to −4.49]) were each associated with a lower EQ-VAS. Finding social distancing easy, compared with neutral (neither carefree nor worried), was associated with a higher EQ-VAS (difference 2.82 [95%CI: 0.21 to 5.44]) in multivariable analysis (Table 2 for multivariable analysis and Table S6, Supplemental Digital Content 3, http://links.lww.com/QAI/B932 for univariable analysis). HIV status was not independently associated with EQ-VAS. There was no significant interaction with HIV status for the association of being worried about getting ill with COVID-19 and EQ-VAS.

**TABLE 2.** Factors Associated With EQ-VAS in Multivariable Fractional Logistic Regression in 497 Participants of the AGEhIV COVID-19 Substudy in Amsterdam From September 2020 Until November 2020

	Difference^A^ [95%CI]	P
How worried are you about getting ill with COVID-19?		**<0.001**
Not worried	0.46 [−2.25 to 3.17]	
Neutral	Ref.	
Worried	−5.15 [−7.92 to −2.38]	
What is your opinion on social distancing?		**0.042**
Difficult	−1.46 [−4.53 to 1.61]	
Neutral	Ref.	
Easy	2.82 [0.21 to 5.44]	
HIV status		0.335
Negative	Ref.	
Positive	−1.11 [−3.38 to 1.15]	
Number of comorbidities ^B^		**<0.001**
0	Ref.	
1–2	−1.16 [−3.40 to 1.08]	
3–7	−10.02 [−15.55 to −4.49]	

2 of 499 participants with missing data were excluded from this analysis.^A^ Difference in EQ-VAS compared with the reference group;^B^ Last available data before the start of the COVID-19 pandemic (February 2020).

Ref, Reference.

### Association Between Social Distancing and Clinically Relevant Depressive Symptoms

Participants who answered that they were worried about getting ill with COVID-19 had a significantly higher odds of clinically relevant depressive symptoms (OR 4.76 [95%CI: 2.01 to 11.30]) than those who answered “neutral’ to this question in multivariable analysis (Table 3 for multivariable analysis and Table S7, Supplemental Digital Content 3, http://links.lww.com/QAI/B932 for univariable analysis). Participants who were 65 years or older, compared with those younger than 60 years, had lower odds of clinically relevant depressive symptoms (OR for 65–69 years 0.23 [95%CI: 0.07 to 0.79]; OR for ≥70 years 0.25 [95%CI: 0.07 to 0.86]). HIV status was not associated with clinically relevant depressive symptoms. There was no significant interaction with HIV status for the association between being worried about getting COVID-19 and clinically relevant depressive symptoms.

**TABLE 3.** Factors Associated With Clinically Relevant Depressive Symptoms in Multivariable Logistic Regression Analysis in 498 Participants of the AGEhIV COVID-19 Substudy in Amsterdam From September 2020 Until November 2020

	Odds Ratio [95% CI]	P
How worried are you about getting ill with COVID-19?		**0.001**
Not worried	2.19 [0.87 to 5.50]	
Neutral	Ref.	
Worried	4.76 [2.01 to 11.30]	
HIV status		0.707
Negative	Ref.	
Positive	0.88 [0.45 to 1.71]	
Age, y		**0.028**
<60	Ref.	
60–64	0.70 [0.32 to 1.54]	
65–69	0.23 [0.07 to 0.79]	
≥70	0.25 [0.07 to 0.86]	

1 of 499 participant with missing data was excluded from this analysis.

Ref, Reference.

### Post hoc Analysis Using Latent Classes of Social Distancing

In a *post hoc* analysis using LCA, 3 social distancing classes were identified. Individuals belonging to class 1 were those who were not worried about contracting COVID-19, adhered poorly to social distancing, and found it difficult to practice social distancing (n = 165, 33.1%); class 2 were those who were worried about contracting COVID-19, had good adherence to social distancing, but found it difficult to practice social distancing (n = 45, 9.0%); and class 3 were those who were worried about contracting COVID-19, had good adherence to social distancing, and found it easy to practice social distancing (n = 289, 57.9%). The distributions of each variable used to establish classes are summarized per class in Table S8, Supplemental Digital Content 3, http://links.lww.com/QAI/B932. In multivariable analysis, there was no significant association between social distancing class and EQ-VAS or between social distancing class and clinically relevant depressive symptoms (Table S9, Supplemental Digital Content 3, http://links.lww.com/QAI/B932). Furthermore, no significant association or interaction with HIV status was found in either of these analyses.

## DISCUSSION

During the initial phase of the COVID-19 pandemic in the Netherlands, a similar majority of HIV-positive and HIV-negative cohort participants reported adhering to social distancing, with similarly smaller proportions reporting an increase in substance use and evidence of clinically relevant depressive symptoms. Regardless of HIV status, concerns about getting ill with COVID-19 negatively affected participants' perceived current health and increased their risk of clinically relevant depressive symptoms. No difference was found in experience with and adherence to social distancing between HIV-positive and HIV-negative participants of similar age, educational level, and ethnic origin.

Most of the participants reported no change in their use of tobacco, alcohol, and recreational drugs since the start of the COVID-19 pandemic. Contrary to our expectation, less than 5% reported an increase in smoking or recreational drugs use. This might be due to clubs, restaurants, and coffee shops (ie, legal places in the Netherlands where you can buy cannabis for personal use) being closed.^1^ By contrast, more HIV-positive participants reported drinking alcohol more or more frequently, compared with HIV-negative participants. Whereas alcohol use was somewhat more common in HIV-negative participants before the COVID-19 pandemic, significantly more HIV-positive participants reported increased or more frequent alcohol use during the pandemic. A study by Diaz-Martinez et al^12^ in HIV-positive and HIV-negative participants of the Miami Adult Studies on HIV (MASH) cohort found that anxiety symptoms were positively correlated with excessive drinking during the COVID-19 pandemic. Because we did not use a formally validated tool for assessing anxiety in our study, we were unable to determine whether our HIV-positive participants may have experienced a greater increase in anxiety, potentially explaining the greater increase in alcohol use during the COVID-19 pandemic compared with the HIV-negative participants.

Regarding HRQoL, HIV-positive participants reported to have more problems in the domain mobility and had a slightly lower EQ-VAS than HIV-negative participants. The lower score regarding mobility in the EQ-6D might be due to more age-related comorbidities in PLWH compared with HIV-negative individuals.^6^ Prepandemic studies have previously described lower HRQoL in PLWH.^13^ Thus, the observed differences in HRQoL between HIV-positive and HIV-negative participants in our study might not be related to the COVID-19 pandemic, but rather to other factors, including a difference in burden of comorbidities.

Being worried about contracting SARS-CoV-2 and having ≥3 comorbidities, but not HIV status, were associated with lower EQ-VAS in our current analysis. Finding social distancing easy was associated with higher EQ-VAS. Two studies from Hong Kong and Switzerland also found that people who were worried about getting COVID-19 had a lower HRQoL.^14,15^ Similar to our findings, Duay et al^15^ found no association between adherence to social distancing and HRQoL.

In our study, no significant difference was found in depressive symptoms between HIV-positive and HIV-negative participants. By contrast, Cooley et al^5^ found a significantly higher depression score (Beck Depression Inventory II score) in participants with HIV, compared with HIV-negative participants, early in the COVID-19 pandemic. However, in that study from the United States, HIV-positive participants were more often under financial stress, from disadvantaged areas, and had poorer access to health care compared with the HIV-negative control group,^5^ which could explain this difference. Because we did not collect information on participants' financial circumstances, we were unable to account for its potential impact on our main outcomes.

Being worried about getting ill with SARS-CoV-2, irrespective of HIV status, was associated with a higher odds and 65 years and older with a lower odds of clinically relevant depressive symptoms. Similar to our results, Marroquín et al found in the general population that older age was associated with lower depression scores during the pandemic,^16^ and another study found an association between being worried and higher scores on self-reported depressive symptoms in Chinese individuals and members of their network (ie, families, classmates, neighbors, and colleagues) who had not been quarantined.^17^ Although a few studies reported an association between adherence to social distancing and depressive symptoms in the general population,^16,18,19^ we did not observe such an association.

Although various patterns were observed in the LCA, none of the identified social distancing classes were associated with EQ-VAS or clinically relevant depressive symptoms.

Our study has some limitations. First, this was a cross-sectional analysis, which does not allow a more dynamic view of the impact of social distancing measures before and during the COVID-19 pandemic on HRQoL and clinically relevant depressive symptoms in HIV-positive and HIV-negative participants. Second, women, those of non-White origin and those with lower educational level were relatively underrepresented in our COVID-19 substudy, which may have biased the self-reported attitudes toward social distancing and COVID-19. Moreover, some of the data, such as the number of comorbidities, were obtained from the last available study visit before the pandemic. Because the time since this last visit until the start of the COVID-19 pandemic (February 27, 2020) was a median of 740 (IQR 305–976) days, some participants might in the interim have developed additional comorbidities which could not be accounted for in the analyses. Finally, the results are not necessarily applicable to HIV-positive individuals in general because they were obtained from largely middle-aged and older, more highly educated, mostly White male individuals, with well-controlled HIV-infection, and good access to health care. Future studies representative of PLWH in other parts of the world should endeavor to include more women as well as younger individuals and as much as possible use standardized measures to aid in cross-study comparisons.

In conclusion, self-reported experiences with and adherence to social distancing were largely comparable, as were changes in substance use, between individuals with or without HIV during the initial phase of the COVID-19 pandemic in the Netherlands. Irrespective of HIV status, concerns about getting ill with COVID-19 had a negative impact on individuals' current perceived health, including mental health in increasing their odds of experiencing clinically relevant levels of depressive symptoms. As the COVID-19 pandemic evolves, social distancing measures may on occasion need to be reinstated and the experiences with and adherence to these measures might change. Longitudinal research will therefore be needed to assess this, including the long-term impact on mental health and the needs for mental health support.

## Supplementary Material

SUPPLEMENTARY MATERIAL
