# Supplementary material for: Change in Substance Use and the Effects of Social Distancing on Health-Related Quality of Life and Depressive Symptoms During the COVID-19 Pandemic in People Living With and Without HIV
Source: J Acquir Immune Defic Syndr. 2022 Jul 13;91(3):261–8. doi: 10.1097/QAI.0000000000003055 (PMC9561239; doi:10.1097/QAI.0000000000003055)
Supplement: SUPPLEMENTARY MATERIAL [file qai-91-261-s001.docx]

**Supplementary Digital Content 1.** Questionnaire study visit 1 for participants of the AGE_h_IV COVID-19 substudy

**Questionnaire**

**COVID-19 Sub study**

**DEMOGRAPHIC INFORMATION**

1. **What is your study number?**

………………………………………. (this is your M or P number)

1. **What is your user number?**

……………………………………… (this is your AMC or GGD number)

1. **What are the four digits of your postal code?**

……………………………………….

1. **What is today’s date?**

………/………/…………….. *(dd/mm/yyyy)*

**DIAGNOSIS COVID-19**

*The following questions are about COVID-19. This is the disease caused by the novel coronavirus SARS-CoV-2.*

1. **Have you been diagnosed with coronavirus/COVID-19?**

- No
- Yes

1. **Have you been tested for the new coronavirus?**

- No >> continue with question 12
- Yes >> continue with question 7

1. **When were you tested? (if you are not sure about the date, please make sure to fill out the month you were tested)**

………/………/…………….. *(dd/mm/yyyy)*

1. **Who executed the test?**

- The general practitioner/GP
- The public health service/GGD
- The hospital
- Other, namely …………………………………………………
- I don’t know

1. **Which test was performed?**

- A throat/nasal swab
- A blood test for coronavirus antibodies
- A saliva test
- I took an at-home finger prick self-test
- Other, namely …………………………………………………
- I don’t know

1. **What was your test result?**

- Positive (meaning you have been diagnosed with coronavirus)

>> continue with question 11

- Negative (meaning you have **not** been diagnosed with coronavirus)

>> continue with question 12

- I don’t know

>> continue with question 12

1. **In the 14 days before I was diagnosed with coronavirus/COVID-19 …**

| I was in close contact with someone with (suspected) coronavirus/COVID-19* | 0 Yes | 0 No | 0 I don’t know | Estimated date of last contact:  ………/………/……………..  *(dd/mm/yyyy)* |
| --- | --- | --- | --- | --- |
| I have been in a healthcare facility (including if for my own work) where COVID-19 patients resided | 0 Yes | 0 No | 0 I don’t know | Last date of presence in facility:  ………/………/……………..  *(dd/mm/yyyy)* |

** “Close contact” is defined as “exposure to COVID-19 in a health care facility; working in close proximity to one another or in the same room; traveling together in a car, bus, train, airplane or by other means of transportation; or living together in the same household.”*

**EXPERIENCED SYMPTOMS**

1. **Since March 2020, have you experienced one or more of the following symptoms?**

| Fever (temperature  > 38.5°C) | 0 Yes | 0 No | 0 I don’t know |  |
| --- | --- | --- | --- | --- |
| Chills / rigors | 0 Yes | 0 No | 0 I don’t know |  |
| Runny nose / nasal congestion | 0 Yes | 0 No | 0 I don’t know |  |
| Ear pain | 0 Yes | 0 No | 0 I don’t know |  |
| Cough | 0 Yes | 0 No | 0 I don’t know |  |
| Coughing up phlegm | 0 Yes | 0 No | 0 I don’t know |  |
| Coughing up bloody phlegm | 0 Yes | 0 No | 0 I don’t know |  |
| Sore throat | 0 Yes | 0 No | 0 I don’t know |  |
| Shortness of breath | 0 Yes | 0 No | 0 I don’t know |  |
| Loss of smell | 0 Yes | 0 No | 0 I don’t know |  |
| Loss of taste | 0 Yes | 0 No | 0 I don’t know |  |
| Fatigue / loss of energy | 0 Yes | 0 No | 0 I don’t know |  |
| Muscle ache | 0 Yes | 0 No | 0 I don’t know |  |
| Headache | 0 Yes | 0 No | 0 I don’t know |  |
| Confusion | 0 Yes | 0 No | 0 I don’t know |  |
| Nausea | 0 Yes | 0 No | 0 I don’t know |  |
| Vomiting | 0 Yes | 0 No | 0 I don’t know |  |
| Abdominal pain | 0 Yes | 0 No | 0 I don’t know |  |
| Diarrhoea | 0 Yes | 0 No | 0 I don’t know |  |
| Skin rash | 0 Yes | 0 No | 0 I don’t know |  |
| Chest pain | 0 Yes | 0 No | 0 I don’t know |  |
| Other symptoms | 0 Yes | 0 No | 0 I don’t know | specify  ……………………………… |

1. **Have you experienced multiple episodes of symptoms reported in question 12 since March 2020?**

- No
- Yes

1. **In which month or months have you experienced such above mentioned symptoms?**

………………………………………………………………………………………..

**CONTACT WITH HEALTHCARE PROVIDER**

1. **Since March 2020, have you contacted a healthcare provider regarding symptoms potentially related to coronavirus/COVID-19?**

- No
- Yes, I have been in contact with …
  - The general practitioner or his/her assistant
  - A nurse or assistant of the outpatient clinic
  - The public health service / GGD
  - A specialist/doctor in the hospital; name of specialist and name of the hospital

……………………………………………………………………………..

- - The emergency department (SEH) of the hospital; name of the hospital ……………………………
  - Another healthcare provider or service, namely ……………………………
  - Other, namely ………………………………………

1. **Since March 2020, have you been admitted to the hospital because of COVID-19?**

- No
- Yes; name of the hospital ……………………………………

**CHANGES IN SUBSTANCE USE**

***Smoking of tobacco***

1. **Since March 2020, has there been a change in your smoking behaviour?**

- Yes, I smoke more or more often
- Yes, I smoke less or less often
- Yes, I quit smoking
- No, no change in my smoking behaviour / I never smoke

1. **Have you smoked during the last 6 months?**

- No >> continue with question 20
- Yes >> continue with question 19

1. **How many cigarettes a day do you smoke on average? (during the last 6 months)**

…………………………………………

***Use of alcohol***

1. **Since March 2020, has there been a change in your use of alcohol?**

- Yes, I drink alcohol more or more often
- Yes, I drink alcohol less or less often
- Yes, I quit drinking alcohol
- No, my alcohol consumption has not changed / I never drink alcohol

1. **Have you drunk alcohol during the past 6 months?**

- No >> continue with question 24
- Yes >> continue with question 22

1. **How often do you drink alcoholic beverages? (during the last 6 months)**

- Less than monthly
- Monthly
- Weekly
- Daily or almost daily

*With ‘one alcohol unit’ we mean a standardized amount of alcohol of approximately 10 grams pure alcohol. This is, for example, a glass of 250mL beer (5%), a glass of 100mL wine (12%) or a shot glass of 35mL strong liquor (35%).*

1. **How many alcohol units do you drink on average on a typical alcohol-consuming day? (during the last 6 months)**

- 1-2
- 3-4
- 5-6
- 7-9
- 10 or more

***Recreational drug use***

*Recreational drugs are for example: Hashish/Weed, Poppers, XTC/MDMA, Cocaine, 4-FA/4-FMP, Amphetamines (Speed/Pep), GHB/GBL, Ketamine, Mephedrone (MiauwMiaw/4-MMC), Methylamphetamine(Tina/Crystal meth), Mushrooms, Heroin, 2-CB or LSD.*

1. **Since March 2020, has there been a change in your use of recreational drugs?**

- Yes, I use drugs more or more often
- Yes, I use drugs less or less often
- Yes, I quit using drugs
- No, no change in my use of drugs / I never use drugs

1. **Have you used recreational drugs during the last 6 months?**

- No >> continue with question 29
- Yes >> continue with question 26

1. **Indicate if you have used any of the following drugs during the last 6 months and if yes, how often and possibly during sex.**

*A steady partner is someone you’re in a steady (sexual) relationship with. A casual partner is someone you’re not in a steady relationship with. Sex means vaginal and/or anal sex, so it doesn’t include oral sex.*

| **Drug** | **Used?** | **If yes, how often?** | **Have you had sex under the influence of this drug with a steady partner?** | **Have you had sex under the influence of this drug with a casual partner?** |
| --- | --- | --- | --- | --- |
| Hashish/Weed (Cannabis, stickie, Hash(oil), THC) | 0 Yes  0 No | 0 Daily  0 Weekly  0 Monthly  0 Sporadically | 0 Yes  0 No  0 Unknown | 0 Yes  0 No  0 Unknown |
| XTC/MDMA | 0 Yes  0 No | 0 Daily  0 Weekly  0 Monthly  0 Sporadically | 0 Yes  0 No  0 Unknown | 0 Yes  0 No  0 Unknown |
| Cocaine/Coke | 0 Yes  0 No | 0 Daily  0 Weekly  0 Monthly  0 Sporadically | 0 Yes  0 No  0 Unknown | 0 Yes  0 No  0 Unknown |
| Amphetamine  (Speed, Pep) | 0 Yes  0 No | 0 Daily  0 Weekly  0 Monthly  0 Sporadically | 0 Yes  0 No  0 Unknown | 0 Yes  0 No  0 Unknown |
| GHB/GBL | 0 Yes  0 No | 0 Daily  0 Weekly  0 Monthly  0 Sporadically | 0 Yes  0 No  0 Unknown | 0 Yes  0 No  0 Unknown |
| Ketamine (special K) | 0 Yes  0 No | 0 Daily  0 Weekly  0 Monthly  0 Sporadically | 0 Yes  0 No  0 Unknown | 0 Yes  0 No  0 Unknown |
| Mephedrone (MiauwMiauw, 4-MMC) | 0 Yes  0 No | 0 Daily  0 Weekly  0 Monthly  0 Sporadically | 0 Yes  0 No  0 Unknown | 0 Yes  0 No  0 Unknown |
| Methylamfetamine (Tina, Crystal Meth, Ice, Glass) | 0 Yes  0 No | 0 Daily  0 Weekly  0 Monthly  0 Sporadically | 0 Yes  0 No  0 Unknown | 0 Yes  0 No  0 Unknown |
| Other drugs, namely  …………………………  ………………………… | 0 Yes  0 No | 0 Daily  0 Weekly  0 Monthly  0 Sporadically | 0 Yes  0 No  0 Unknown | 0 Yes  0 No  0 Unknown |

1. **Since March 2020, have you injected any recreational drugs?**

- Yes >> continue with question 28
- No >> continue with question 29

1. **How often did you inject recreational drugs during the last 6 months?**

- Daily
- Weekly
- Monthly
- Less than monthly

**CHANGE IN SEXUAL BEHAVIOUR**

*The following questions are about your sexual behaviour since the start of the coronavirus pandemic in March 2020.*

*A casual partner is someone you’re not in a steady relationship with. Sexual intercourse means vaginal and/or anal sex, so it doesn’t include oral sex.*

1. **Did you have sexual intercourse with casual partners more or less often since March 2020?**

- Yes, I have had sexual intercourse with casual partners more often
- Yes, I have had sexual intercourse with casual partners less often
- No, there has been no change in the frequency of sexual intercourse with casual partners

1. **How many different men did you have sexual intercourse with during the last 6 months?**

………………………………………………………….

1. **How many different women did you have sexual intercourse with during the last 6 months?**

………………………………………………………….

1. **Do you have HIV?**

- No >> continue with question 33
- Yes >> continue with question 36

**CHANGE IN USE OF PrEP**

*PrEP means pre-exposure prophylaxis and contains anti-HIV medication. This medication is used to prevent you from becoming HIV infected..*

1. **Since March 2020, have you used PrEP?**

- No
- Yes

1. **Since March 2020, has there been a change your PrEP use?**

- Yes, I have used PrEP more, or more often
- Yes, I have used PrEP less, or less often
- No, I did not change my PrEP use or have never used PrEP

1. **Since March 2020, has your ability to obtain PrEP been impacted?**

- Yes, it has become more difficult to obtain PrEP
- No, it has not become more difficult to obtain PrEP
- I have not tried to obtain PrEP

*Continue with question 40*

**CHANGE IN USE OF HIV MEDICATION**

1. **Are you currently taking medication to treat your HIV?**

- Yes
- No

1. **During the past week, how many days did you take your HIV medication?**

- 0 days a week
- 1 days a week
- 2 days a week
- 3 days a week
- 4 days a week
- 5 days a week
- 6 days a week
- 7 days a week

1. **Since March 2020, has the way you take your HIV medication changed?**

- I forgot to take my HIV medication less often
- I forgot to take my HIV medication more often
- Nothing has changed in the way I take my HIV medication

1. **Since March 2020, has your ability to get your HIV medication been impacted?**

- Yes, it has become more difficult to get my HIV medication
- No, it has not become more difficult to get my HIV medication

**CHANGE IN SOCIAL BEHAVIOUR**

*The following questions are about the possible changes in your behaviour as result of the coronavirus pandemic and the measures implemented by the government since March 2020. With ‘social distancing’ we mean limiting contact between people to avoid spreading of an infectious disease, for instance by staying at home as much as possible, avoiding unnecessary contact with others, keeping 1.5 meter distance to others and avoiding gatherings with three or more adults.*

1. **How important is it to you to prevent getting infected with the new coronavirus?**

Very unimportant  1  2  3  4  5  6  7 Very important

1. **How worried are you about getting sick with COVID-19?**

Not worried at all  1  2  3  4  5  6  7 Very worried

1. **How well have you generally complied with the ‘social distancing’ measures?**

Very poorly  1  2  3  4  5  6  7 Very well

1. **What is your opinion on ‘social distancing’?**

Very unimportant  1  2  3  4  5  6  7 Very important

Very hard to do  1  2  3  4  5  6  7 Very easy to do

1. **How many persons (including yourself) does your household comprise of?**

- I live alone >> continue with question 46
- My household comprises of ………… persons >> continue with question 45

1. **How well have the adults in your household generally been able to comply with the ‘social distancing’ measures?**

Very poorly  1  2  3  4  5  6  7 Very well

**QUALITY OF LIFE**

*Under each heading, please tick the ONE box that best describes your health TODAY.*

1. **MOBILITY**

- I have no problems in walking about
- I have slight problems in walking about
- I have moderate problems in walking about
- I have severe problems in walking about
- I am unable to walk about

1. **SELF-CARE**

- I have no problems washing or dressing myself
- I have slight problems washing or dressing myself
- I have moderate problems washing or dressing myself
- I have severe problems washing or dressing myself
- I am unable to wash or dress myself

1. **USUAL ACTIVITIES (e.g. work, study, housework, family or leisure activities)**

- I have no problems doing my usual activities
- I have slight problems doing my usual activities
- I have moderate problems doing my usual activities
- I have severe problems doing my usual activities
- I am unable to do my usual activities

1. **PAIN / DISCOMFORT**

- I have no pain or discomfort
- I have slight pain or discomfort
- I have moderate pain or discomfort
- I have severe pain or discomfort
- I have extreme pain or discomfort

1. **ANXIETY / DEPRESSION**

- I am not anxious or depressed
- I am slightly anxious or depressed
- I am moderately anxious or depressed
- I am severely anxious or depressed
- I am extremely anxious or depressed

1. **COGNITION**

- I have no problems with cognitive functioning (e.g. memory, concentration).
- I have some problems with cognitive functioning (e.g. memory, concentration).
- I have extreme problems with cognitive functioning (e.g. memory, concentration).

1. **SCALE**


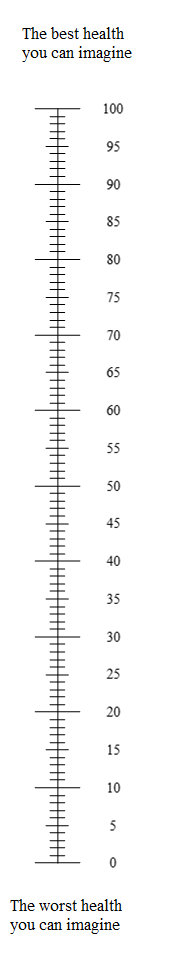
We would like to know how good or bad your health is *TODAY*. This scale is numbered from 0 to 100.

100 means the best health you can imagine.

0 means the worst health you can imagine.

Mark an X on the scale to indicate how your health is TODAY.

Now, please write the number you marked on the scale in the box below.

**DEPRESSION**

*Over the last 2 weeks, how often have you been bothered by any of the following items?*

1. **Little interest or pleasure in doing things**

- Not at all
- Several days
- More than half the days
- Nearly every day

1. **Feeling down, depressed or hopeless**

- Not at all
- Several days
- More than half the days
- Nearly every day

1. **Trouble falling asleep, staying asleep, or sleeping too much**

- Not at all
- Several days
- More than half the days
- Nearly every day

1. **Feeling tired or having little energy**

- Not at all
- Several days
- More than half the days
- Nearly every day

1. **Poor appetite or overeating**

- Not at all
- Several days
- More than half the days
- Nearly every day

1. **Feeling bad about yourself – or that you’re a failure or have let yourself or your family down**

- Not at all
- Several days
- More than half the days
- Nearly every day

1. **Trouble concentrating on things, such as reading the newspaper or watching television**

- Not at all
- Several days
- More than half the days
- Nearly every day

1. **Moving or speaking so slowly that other people could have noticed. Or the opposite – being so fidgety or restless that you have been moving around a lot more than usual**

- Not at all
- Several days
- More than half the days
- Nearly every day

1. **Thoughts that you would be better off dead or of hurting yourself in some way**

- Not at all
- Several days
- More than half the days
- Nearly every day

**COMMENTS**

1. **This is the final question of this questionnaire, please provide any comments here related to this questionnaire or to the AGE_h_IV** **study.**

*Please do not provide any personal contact details.*

…………………………………………………………………………………………

…………………………………………………………………………………………

…………………………………………………………………………………………

…………………………………………………………………………………………

…………………………………………………………………………………………

…………………………………………………………………………………………

…………………………………………………………………………………………

…………………………………………………………………………………………

…………………………………………………………………………………………

…………………………………………………………………………………………

…………………………………………………………………………………………

***THE END***

***Thank you very much for your participation and for completing this questionnaire!***

***The AGE_h_IV*** ***studyteam***
