# Supplementary material for: Change in Substance Use and the Effects of Social Distancing on Health-Related Quality of Life and Depressive Symptoms During the COVID-19 Pandemic in People Living With and Without HIV
Source: J Acquir Immune Defic Syndr. 2022 Jul 13;91(3):261–8. doi: 10.1097/QAI.0000000000003055 (PMC9561239; doi:10.1097/QAI.0000000000003055)
Supplement: SUPPLEMENTARY MATERIAL [file qai-91-261-s002.docx]

**Supplementary Digital Content 2.** Flow diagram included and excluded participants

Assessed for eligibility
(in active follow-up in the AGE_h_IV cohort study and living (at least partly)in the Netherlands)

n=824
(378 HIV-positive and 446 HIV-negative participants)

Completed the study questionnaire
n=512
(218 HIV-positive and 294 HIV-negative participants)

Excluded from analysis due to reporting positive COVID-19 test or diagnosed with SARS-CoV-2 infection (n=13)

Did not complete the study questionnaire (n=38)

Not included (n=274)

- Declined to participate (n=174)
- Did not respond to invitations (n=62)
- Unable to participate in September/October 2020 (n=38)

Included in analysis
n=499
(214 HIV-positive and 285 HIV-negative participants)

Included in the COVID-19 substudy
n=550
(238 HIV-positive and 312 HIV-negative participants)
