# Supplementary material for: Change in Substance Use and the Effects of Social Distancing on Health-Related Quality of Life and Depressive Symptoms During the COVID-19 Pandemic in People Living With and Without HIV
Source: J Acquir Immune Defic Syndr. 2022 Jul 13;91(3):261–8. doi: 10.1097/QAI.0000000000003055 (PMC9561239; doi:10.1097/QAI.0000000000003055)
Supplement: SUPPLEMENTARY MATERIAL [file qai-91-261-s003.docx]

**Supplementary Digital Content 3**. Supplementary tables S1-S9

**Supplementary Table S1**. Distribution of 7-point Likert scale for social distancing in the AGE_h_IV COVID-19 substudy, in Amsterdam from September 2020 until November 2020, by HIV-status

|  | **HIV-positive participants  (n = 214) Median (IQR)** | **HIV-negative participants (n = 285) Median (IQR)** |
| --- | --- | --- |
| *How important is it to you to prevent getting infected with the new coronavirus?* | 7 (6-7) | 7 (6-7) |
| *How worried are you about getting ill with COVID-19?* | 4 (3-6) | 5 (3-6) |
| *How well have you generally complied with the ‘social distancing’ measures?* | 6 (5-7) | 6 (5-6) |
| *What is your opinion on social distancing? (Importance)* | 7 (6-7) | 6 (6-7) |
| *What is your opinion on social distancing? (Difficulty)* | 5 (4-6) | 5 (4-6) |
| *How well have the adults in your household generally been able to comply with the social distancing measures? ^A^* | 6 (5-7) | 6 (5-7) |

^A^ Only in participants with household members besides the index (n= 273 participants; 121 HIV-positive participants, 152 HIV-negative participant)
Abbreviations: HIV, Human Immunodeficiency Virus; IQR, Interquartile Range.

**Supplementary Table S2.** Characteristics of the AGE_h_IV COVID-19 substudy participants included in the current analysis and of those who declined substudy participation or who were excluded from the current analysis, in Amsterdam from September 2020 until November 2020

|  | **Included participants  (n = 499) No. (%) or median (IQR)** | **Declining or excluded participants  (n = 325) No. (%) or median (IQR)** | **P** |
| --- | --- | --- | --- |
| **Demographics** | | | |
| *Self-identified female gender* | 64 (12.8) | 48 (14.8) | 0.426^*^ |
| *Age, y ^A^* |  |  |  |
| <60 | 229 (45.9) | 144 (44.3) | 0.471^*^ |
| 60-64 | 117 (23.5) | 80 (24.6) |  |
| 65-69 | 83 (16.6) | 45 (13.9) |  |
| ≥70 | 70 (14.0) | 56 (17.2) |  |
| *HIV-positive* | 214 (42.9) | 168 (51.7) | **0.013^*^** |
| *Ethnic origin* |  |  | **0.001^*^** |
| Caucasian | 475 (95.2) | 286 (88.0) |  |
| African | 17 (3.4) | 31 (9.5) |  |
| Asian | 7 (1.4) | 8 (2.5) |  |
| *Educational level ^B,C^* |  |  | **0.005^*^** |
| Lower education | 211 (42.5) | 162 (52.1) |  |
| Higher education | 277 (55.9) | 139 (44.7) |  |
| Other | 8 (1.6) | 10 (3.2) |  |
| *Number of comorbidities ^D^* |  |  | 0.150^*^ |
| 0 | 269 (53.9) | 154 (47.4) |  |
| 1-2 | 194 (38.9) | 140 (43.1) |  |
| 3-7 | 36 (7.2) | 31 (9.5) |  |
| **Behavioral characteristics ^D,E^** | | | |
| *Cigarette smoking ^F^* | 102 (20.4) | 83 (25.7) | 0.078^*^ |
| *Alcohol use ^G^* | 418 (83.8) | 254 (78.6) | 0.063^*^ |
| *Recreational drugs use* | 163 (34.0) | 82 (31.7) | 0.527^*^ |

Missing data were not considered in the comparisons.
^A^ Age at the time of being invited to the substudy on August 1, 2020; ^B^ Data from the first study round of the AGE_h_IV study; ^C^ Missing data from 3 (0.6%) included participants and 14 (4.3%) excluded participants; ^D^ Last available data prior to the start of the COVID-19 pandemic (February 2020); ^E^ In the six months before answering the question; ^F^ Missing data from 2 (0.6%) excluded participants; ^G^ Missing data from 19 (3.8%) included participants and 66 (20.3%) excluded participants
^*^Pearson’s χ^2^
Abbreviations: HIV, Human Immunodeficiency Virus; IQR, Interquartile Range; No, Number; P, P-value; y, years

**Supplementary Table S3.** Experience with and adherence to social distancing measures in participants of the AGE_h_IV COVID-19 substudy, by HIV status, in Amsterdam from September 2020 until November 2020

|  | **HIV-positive participants  (n = 214) No. (%) or median (IQR)** | **HIV-negative participants (n = 285) No. (%) or median (IQR)** | **P** |
| --- | --- | --- | --- |
| *How important is it to you to prevent getting infected with the new coronavirus?* |  |  | 0.623 |
| Unimportant (1-3) ^A^ | 14 (6.5) | 22 (7.7) |  |
| Neutral (4-5) | 25 (11.7) | 40 (14.0) |  |
| Important (6-7) | 175 (81.8) | 223 (78.3) |  |
| *How worried are you about getting ill with COVID-19?* |  |  | 0.094 |
| Not worried (1-3) | 69 (32.4) | 81 (28.4) |  |
| Neutral (4-5) | 73 (34.3) | 125 (43.9) |  |
| Worried (6-7) | 71 (33.3) | 79 (27.7) |  |
| *How well have you generally complied with the ‘social distancing’ measures?* |  |  | 0.110 |
| Poorly (1-3) | 11 (5.1) | 13 (4.6) |  |
| Neutral (4-5) | 50 (23.4) | 91 (31.9) |  |
| Well (6-7) | 153 (71.5) | 181 (63.5) |  |
| *What is your opinion on social distancing?* |  |  | 0.294 |
| Unimportant (1-3) | 16 (7.5) | 16 (5.6) |  |
| Neutral (4-5) | 29 (13.6) | 52 (18.3) |  |
| Important (6-7) | 169 (79.0) | 217 (76.1) |  |
| *What is your opinion on social distancing?* |  |  | 0.547 |
| Difficult (1-3) | 44 (20.7) | 56 (19.7) |  |
| Neutral (4-5) | 102 (47.9) | 126 (44.2) |  |
| Easy (6-7) | 67 (31.5) | 103 (36.1) |  |
| *Household size, median ^B^* | 2 (1-2) | 2 (1-2) | 0.469 |
| *How well have the adults in your household generally been able to comply with the social distancing measures? ^C^* |  |  | 0.106 |
| Poorly (1-3) | 17 (14.2) | 14 (9.2) |  |
| Neutral (4-5) | 24 (20.0) | 46 (30.3) |  |
| Well (6-7) | 79 (65.8) | 92 (60.5) |  |

^A^ Between brackets is indicated the range of the Likert scale underlying each category; ^B^ Includes the participants self; ^C^ Only in participants with household members besides the index (n= 273 participants; 121 HIV-positive participants, 152 HIV-negative participant)
Abbreviations: HIV, Human Immunodeficiency Virus; IQR, Interquartile Range; No, Number; P, P-value

**Supplementary Table S4.** Reported change in substance use since the start of the COVID-19 pandemic in participants of the AGE_h_IV COVID-19 substudy, by HIV status, in Amsterdam from September 2020 until November 2020

| **Change in substance use** | **HIV-positive participants (n = 214) No. (%)** | **HIV-negative participants (n = 285) No. (%)** | **P** |
| --- | --- | --- | --- |
| *Change in smoking* |  |  | 0.777 |
| Smoking more or more often | 6 (2.8) | 13 (4.6) |  |
| No change in smoking/never smoked | 183 (86.3) | 242 (85.5) |  |
| Smoking less or less often | 9 (4.3) | 11 (3.9) |  |
| Quit smoking | 14 (6.6) | 17 (6.0) |  |
| *Change in alcohol use* |  |  | **0.005** |
| Drinking more or more often | 26 (12.2) | 14 (4.9) |  |
| No change in drinking/ never drank | 156 (73.2) | 208 (73.2) |  |
| Drinking less or less often | 25 (11.7) | 56 (19.7) |  |
| Quit drinking | 6 (2.8) | 6 (2.1) |  |
| *Change in recreational drug use* |  |  | 0.985 |
| Using drugs more or more often | 5 (2.3) | 7 (2.5) |  |
| No change in using drugs/never used drugs | 187 (87.4) | 244 (86.2) |  |
| Using drugs less or less often drug use | 15 (7.0) | 22 (7.8) |  |
| Quit using drugs | 7 (3.3) | 10 (3.5) |  |

Abbreviations: HIV, Human Immunodeficiency Virus; No., Number; P, P-value

**Supplementary Table S5**. Health-related quality of life assessed by the EQ-6D tool during the COVID-19 pandemic in participants of the AGEhIV COVID-19 substudy, by HIV status, in Amsterdam from September 2020 until November 2020

| **Domains of health-related quality of life** | **HIV-positive participants  (n = 214) No. (%) or median (IQR)** | **HIV-negative participants  (n = 285) No. (%) or median (IQR)** | **P** |
| --- | --- | --- | --- |
| *Mobility* |  |  | **0.011** |
| No problems | 162 (75.7) | 249 (87.4) |  |
| Slight problems | 25 (11.7) | 20 (7.0) |  |
| Moderate problems | 22 (10.3) | 12 (4.2) |  |
| Severe problems | 4 (1.9) | 4 (1.4) |  |
| Unable to | 1 (0.5) | 0 (0.0) |  |
| *Self-care* |  |  | 0.515 |
| No problems | 203 (94.9) | 275 (96.5) |  |
| Slight problems | 10 (4.7) | 8 (2.8) |  |
| Moderate problems | 1 (0.5) | 2 (0.7) |  |
| Severe problems | 0 (0.0) | 0 (0.0) |  |
| Unable to | 0 (0.0) | 0 (0.0) |  |
| *Usual activities* |  |  | 0.105 |
| No problems | 162 (76.1) | 239 (83.9) |  |
| Slight problems | 39 (18.3) | 31 (10.9) |  |
| Moderate problems | 10 (4.7) | 11 (3.9) |  |
| Severe problems | 2 (0.9) | 4 (1.4) |  |
| Unable to | 0 (0.0) | 0 (0.0) |  |
| *Pain* |  |  | 0.065 |
| No pain or discomfort | 111 (52.1) | 177 (62.1) |  |
| Slight pain or discomfort | 67 (31.5) | 81 (28.4) |  |
| Moderate pain of discomfort | 29 (13.6) | 22 (7.7) |  |
| Severe pain or discomfort | 6 (2.8) | 5 (1.8) |  |
| Extreme pain or discomfort | 0 (0.0) | 0 (0.0) |  |
| *Anxiety/depression* |  |  | 0.611 |
| Not anxious or depressed | 140 (65.4) | 169 (59.3) |  |
| Slightly anxious or depressed | 48 (22.4) | 77 (27.0) |  |
| Moderately anxious or depressed | 23 (10.8) | 33 (11.6) |  |
| Severely anxious or depressed | 3 (1.4) | 5 (1.8) |  |
| Extremely anxious or depressed | 0 (0.0) | 1 (0.4) |  |
| *Cognition* |  |  | 0.908 |
| No problems | 156 (72.9) | 204 (71.6) |  |
| Some problems | 57 (26.6) | 79 (27.7) |  |
| Extreme problems | 1 (0.5) | 2 (0.7) |  |
| *EQ-VAS* | 80 (73-90) | 84 (75-90) | **0.041** |

Abbreviations: HIV, Human Immunodeficiency Virus; IQR, Interquartile Range; No, Number; P, P-value

**Supplementary Table S6.** Factors associated with EQ-VAS in univariable fractional logistic regression in participants of the AGE_h_IV COVID-19 substudy in Amsterdam from September 2020 until November 2020

|  | **EQ-VAS** |  | | |
| --- | --- | --- | --- | --- |
|  | **Mean [95%CI]** | | **Difference ^A^ [95%CI]** | **P** |
| *How important is it to you to prevent getting infected with the new coronavirus?* |  | |  | 0.694 |
| Unimportant | 78.69 [72.31 to 85.08] | | -2.38 [-9.02 to 4.26] |  |
| Neutral | 81.08 [78.35 to 83.81] | | Ref. |  |
| Important | 81.29 [79.99 to 82.58] | | 0.21 [-2.75 to 3.16] |  |
| *How worried are you about getting ill with COVID-19?* |  | |  | <0.001 |
| Not worried | 82.84 [80.59 to 85.09] | | 0.36 [-2.27 to 2.99] |  |
| Neutral | 82.48 [81.07 to 83.90] | | Ref. |  |
| Worried | 77.35 [74.81 to 79.88] | | -5.14 [-8.01 to -2.26] |  |
| *How well have you generally complied with the ‘social distancing’ measures?* |  | |  | 0.834 |
| Poorly | 81.33 [75.97 to 86.69] | | 0.78 [-4.54 to 6.10] |  |
| Neutral | 80.55 [78.66 to 82.44] | | Ref. |  |
| Well | 81.27 [79.74 to 82.80] | | 0.72 [-1.69 to 3.13] |  |
| *What is your opinion on social distancing?* |  | |  | 0.122 |
| Unimportant | 84.56 [80.71 to 88.42] | | 2.38 [-2.07 to 6.82] |  |
| Neutral | 82.19 [79.60 to 84.77] | | Ref. |  |
| Important | 80.55 [79.16 to 81.93] | | -1.64 [-4.52 to 1.25] |  |
| *What is your opinion on social distancing?* |  | |  | 0.118 |
| Difficult | 79.23 [76.33 to 82.13] | | -1.34 [-4.56 to 1.89] |  |
| Neutral | 80.57 [79.05 to 82.09] | | Ref. |  |
| Easy | 82.81 [80.60 to 85.02] | | 2.25 [-0.41 to 4.91] |  |
| *How well have the adults in your household generally been able to comply with the social distancing measures?* |  | |  | 0.468 |
| Poorly | 81.48 [77.99 to 84.98] | | -1.89 [-6.03 to 2.26] |  |
| Neutral | 83.37 [80.80 to 85.94] | | Ref. |  |
| Well | 81.39 [79.30 to 83.48] | | -1.99 [-5.24 to 1.27] |  |
| *Household size* |  | |  | 0.105 |
| Alone | 80.00 [78.16 to 81.85] | | Ref. |  |
| With someone | 81.96 [80.45 to 83.47] | | 1.95 [-0.42 to 4.32] |  |
| *HIV status* |  | |  | 0.087 |
| Negative | 81.96 [80.43 to 83.49] | | Ref. |  |
| Positive | 79.89 [78.06 to 81.72] | | -2.07 [-4.44 to 0.31] |  |
| *Self-identified gender* |  | |  | 0.380 |
| Female | 82.54 [79.02 to 86.06] | | 1.68 [-1.97 to 5.33] |  |
| Male | 80.86 [79.61 to 82.11] | | Ref. |  |
| *Age, y* |  | |  | 0.943 |
| <60 | 80.87 [79.01 to 82.74] | | Ref. |  |
| 60-64 | 81.20 [78.84 to 83.55] | | 0.32 [-2.64 to 3.29] |  |
| 65-69 | 81.69 [79.50 to 83.88] | | 0.82 [-2.02 to 3.66] |  |
| ≥70 | 80.72 [77.13 to 84.31] | | -0.15 [-4.12 to 3.81] |  |
| *Ethnic origin* |  | |  | 0.524 |
| Caucasian | 81.11 [79.91 to 82.30] | | Ref. |  |
| African | 82.41 [74.56 to 90.26] | | 1.31 [-5.84 to 8.46] |  |
| Asian | 75.57 [60.83 to 90.31] | | -5.53 [-16.54 to 5.47] |  |
| *Educational level ^B^* |  | |  | 0.460 |
| Lower education | 81.02 [79.01 to 83.03] | | Ref. |  |
| Higher education | 81.37 [79.96 to 82.78] | | 0.35 [-2.09 to 2.79] |  |
| Other | 74.50 [59.03 to 89.97] | | -6.52 [-18.70 to 5.65] |  |
| *Number of comorbidities ^C^* |  | |  | <0.001 |
| 0 | 82.44 [80.91 to 83.97] | | Ref. |  |
| 1-2 | 80.92 [79.15 to 82.69] | | -1.52 [-3.85 to 0.80] |  |
| 3-7 | 71.67 [65.59 to 77.74] | | -10.78 [-16.76 to -4.79] |  |
| *Self-reported change in smoking* |  | |  | 0.481 |
| Smoking more or more often | 77.47 [69.80 to 85.15] | | -3.89 [-10.97 to 3.19] |  |
| No change in smoking/never smoked | 81.37 [80.15 to 82.59] | | Ref. |  |
| Smoking less or less often | 83.25 [77.11 to 89.39] | | 1.88 [-3.85 to 7.62] |  |
| Quit smoking | 78.61 [71.79 to 85.44] | | -2.75 [-9.32 to 3.81] |  |
| *Self-reported change in alcohol use* |  | |  | 0.253 |
| Drinking more or more often | 77.33 [72.86 to 81.79] | | -4.27 [-8.74 to 0.20] |  |
| No change in drinking/never drank | 81.59 [80.27 to 82.91] | | Ref. |  |
| Drinking less or less often | 81.27 [77.99 to 84.55] | | -0.32 [-3.79 to 3.15] |  |
| Quit drinking | 79.58 [69.59 to 89.58] | | -2.01 [-10.64 to 6.62] |  |
| *Self-reported change in recreational drug use* |  |  | | 0.955 |
| Using drugs more or more often | 80.00 [74.07 to 85.93] | -1.13 [-6.35 to 4.09] | |  |
| No change in using drugs/never used drugs | 81.13 [79.84 to 82.42] | Ref. | |  |
| Using drugs less or less often | 81.54 [77.79 to 85.29] | 0.41 [-3.39 to 4.22] | |  |
| Quit using drugs | 80.12 [72.49 to 87.74] | -1.01 [-7.98 to 5.96] | |  |

^A^ Difference in EQ-VAS compared to the reference group; ^B^ Data from the first study round of the AGE_h_IV study; ^C^ Last available data prior to the start of the COVID-19 pandemic (February 2020)
Abbreviations: 95%CI, 95% confidence interval; HIV, Human Immunodeficiency Virus; IQR; interquartile range; P, P-value; Ref, Reference; y, year

**Supplementary Table S7**. Factors associated with clinically-relevant depressive symptoms in univariable logistic regression analysis in participants of the AGE_h_IV COVID-19 substudy in Amsterdam from September 2020 until November 2020

|  | **Clinically-relevant** **depressive symptoms** |  | |
| --- | --- | --- | --- |
|  | **n/N (%)** | **Odds ratio [95% CI]** | **P** |
| *How important is it to you to prevent getting infected with the new coronavirus?* |  |  | 0.133 |
| Unimportant | 6/36 (16.7) | 4.13 [0.97 to 17.67] |  |
| Neutral | 3/65 (4.6) | Ref. |  |
| Important | 34/398 (8.5) | 1.93 [0.58 to 6.48] |  |
| *How worried are you about getting ill with COVID-19?* |  |  | 0.007 |
| Not worried | 13/150 (8.7) | 2.25 [0.91 to 5.59] |  |
| Neutral | 8/198 (4.0) | Ref. |  |
| Worried | 21/150 (14.0) | 3.87 [1.66 to 9.00] |  |
| *How well have you generally complied with the ‘social distancing’ measures?* |  |  | 0.730 |
| Poorly | 1/24 (4.2) | 0.47 [0.06 to 3.77] |  |
| Neutral | 12/141 (8.5) | Ref. |  |
| Well | 30/334 (9.0) | 1.06 [0.53 to 2.14] |  |
| *What is your opinion on social distancing?* |  |  | 0.789 |
| Unimportant | 2/32 (6.3) | 0.83 [0.16 to 4.36] |  |
| Neutral | 6/81 (7.4) | Ref. |  |
| Important | 35/386 (9.1) | 1.25 [0.51 to 3.07] |  |
| *What is your opinion on social distancing?* |  |  | 0.339 |
| Difficult | 12/100 (12.0) | 1.81 [0.82 to 3.98] |  |
| Neutral | 16/228 (7.0) | Ref. |  |
| Easy | 15/170 (8.8) | 1.28 [0.62 to 2.67] |  |
| *How well have the adults in your household generally been able to comply with the social distancing measures?* |  |  | 0.427 |
| Poorly | 0/31 (0.0) | * |  |
| Neutral | 4/70 (5.7) | Ref. |  |
| Well | 15/171 (8.8) | 1.59 [0.51 to 4.96] |  |
| *Household size* |  |  | 0.261 |
| Alone | 23/226 (10.2) | Ref. |  |
| With someone | 20/273 (7.3) | 0.70 [0.37 to 1.31] |  |
| *HIV status* |  |  | 0.887 |
| Negative | 25/285 (8.8) | Ref. |  |
| Positive | 18/214 (8.4) | 0.96 [0.51 to 1.80] |  |
| *Self-identified gender* |  |  | 0.092 |
| Female | 9/63 (14.3) | 1.97 [0.90 to 4.33] |  |
| Male | 34/436 (7.8) | Ref. |  |
| *Age, y* |  |  | 0.089 |
| <60 | 26/222 (11.7) | Ref. |  |
| 60-64 | 11/117 (9.4) | 0.61 [0.34 to 1.09] |  |
| 65-69 | 3/88 (3.4) | 0.54 [0.29 to 1.00] |  |
| ≥70 | 3/72 (4.2) | 0.50 [0.26 to 0.95] |  |
| *Ethnic origin* |  |  | 0.198 |
| Caucasian | 40/475 (8.4) | Ref. |  |
| African | 3/17 (17.7) | 2.33 [0.64 to 8.45] |  |
| Asian | 0/7 (0.0) | * |  |
| *Educational level ^A^* |  |  | 0.885 |
| Lower education | 18/211 (8.5) | Ref. |  |
| Higher education | 22/277 (7.9) | 0.93 [0.48 to 1.77] |  |
| Other | 1/8 (12.5) | 1.53 [0.18 to 13.15] |  |
| *Number of comorbidities ^B^* |  |  | 0.736 |
| 0 | 21/269 (7.8) | Ref. |  |
| 1-2 | 18/194 (9.3) | 1.21 [0.63 to 2.33] |  |
| 3-7 | 4/36 (11.1) | 1.48 [0.48 to 4.57] |  |
| *Self-reported change in smoking* |  |  | 0.274 |
| Smoking more or more often | 4/19 (21.1) | 3.17 [0.99 to 10.09] |  |
| No change in smoking/never smoked | 33/425 (7.8) | Ref. |  |
| Smoking less or less often | 2/20 (10.0) | 1.32 [0.29 to 5.94] |  |
| Quit smoking | 3/31 (9.7) | 1.27 [0.37 to 4.41] |  |
| *Self-reported change in alcohol use* |  |  | 0.576 |
| Drinking more or more often | 5/40 (12.5) | 1.65 [0.60 to 4.54] |  |
| No change in drinking/never drank | 29/364 (8.0) | Ref. |  |
| Drinking less or less often | 8/81 (9.9) | 1.27 [0.56 to 2.88] |  |
| Quit drinking | 0/12 (0.0) | * |  |
| *Self-reported change in recreational drug use* |  |  | 0.243 |
| Using drugs more or more often | 0/12 (0.0) | * |  |
| No change in using drugs/never used drugs | 35/431 (8.1) | Ref. |  |
| Using drugs less or less often | 6/37 (16.2) | 2.19 [0.86 to 5.61] |  |
| Quit using drugs | 2/17 (11.8) | 1.51 [0.33 to 6.87] |  |

* The prevalence of outcome was 0 for this covariable, hence ORs could not be calculated
^A^ Data from the first study round of the AGE_h_IV study; ^B^ Last available data prior to the start of the COVID-19 pandemic (February 2020)
Abbreviations: 95%CI, 95% confidence interval; HIV, Human Immunodeficiency Virus; P, P-value; Ref, Reference; y, year

**Supplementary Table S8**. Distribution of variables in the classes of experience with and adherence to social distancing from participants of the AGE_h_IV COVID-19 substudy in Amsterdam from September 2020 until November 2020

|  | **Definition** | **Class 1**  **Means** | **Class 2**  **Means** | **Class 3**  **Means** |
| --- | --- | --- | --- | --- |
| *How worried are you about getting ill with COVID-19?* | -1 = not worried 0 = neutral 1 = worried | -0.35 | 0.24 | 0.15 |
| *How well have you generally complied with the ‘social distancing’ measures?* | -1 = poor 0 = neutral 1 = well | -0.15 | 1.00 | 1.00 |
| *What is your opinion on social distancing?* | -1 = difficult 0 = neutral 1 = easy | -0.26 | -0.61 | 0.58 |

Interpretation:
Participants belonging to Class 1 were not worried about getting ill with COVID-19 (-0.35); complied generally poor with the social distancing measures (-0.15); and found social distancing difficult (-0.26)
Participants belonging to Class 2 were somewhat worried about getting ill with COVID-19 (0.24); complied generally well with the social distancing measures (1.00); and found social distancing difficult (-0.61)
Participants belonging to Class 3 were somewhat worried about getting ill with COVID-19 (0.15); complied generally well with the social distancing measures (1.00); and found social distancing easy (0.58)

**Supplementary Table S9**. Association between social distancing class and EQ-VAS in multivariable fractional logistic regression and association between social distancing class and indication of clinically-relevant levels of depressive symptoms in multivariable logistic regression analysis in participants of the AGE_h_IV COVID-19 substudy in Amsterdam from September 2020 until November 2020

|  | **Multivariable fractional logistic regression (EQ-VAS)** | | **Multivariable logistic regression (clinically-relevant depressive symptoms)** | |
| --- | --- | --- | --- | --- |
|  | **Difference ^A^ [95%CI]** | **P** | **Odds ratio [95%CI]** | **P** |
| *Latent classes social distancing* |  | 0.514 |  | 0.755 |
| 1: not worried about contracting COVID-19, poor adherence and difficult to social distance | Ref. |  | Ref. |  |
| 2: being worried about contracting COVID-19, good adherence and difficult to social distance | -0.72 [-5.28 to 3.85] |  | 1.46 [0.44 to 4.84] |  |
| 3: being worried about contracting COVID-19, good adherence and easy to social distance | 1.19 [-1.18 to 3.56] |  | 1.25 [0.62 to 2.53] |  |
| *HIV status* |  | 0.208 |  | 0.944 |
| Negative | Ref. |  | Ref. |  |
| Positive | -1.47 [-3.76 to 0.82] |  | 1.02 [0.54 to 1.95] |  |
| *Number of comorbidities ^B^* |  | <0.001 |  |  |
| 0 | Ref. |  | - |  |
| 1-2 | -1.44 [-3.79 to 0.91] |  | - |  |
| 3-7 | -10.49 [-16.35 to -4.63] |  | - |  |
| *Age ^C^, y* |  |  |  | 0.064 |
| <60 | - |  | Ref. |  |
| 60-64 | - |  | 0.77 [0.37 to 1.63] |  |
| 65-69 | - |  | 0.25 [0.07 to 0.87] |  |
| ≥70 | - |  | 0.32 [0.09 to 1.08] |  |

^A^ Difference in EQ-VAS compared to the reference group; ^B^ Comorbidities were not associated in univariable analysis with PHQ-9 and thus not included in multivariable analysis with PHQ-9; ^C^ Age was not associated in univariable analysis with EQ-VAS and thus not included in multivariable analysis with EQ-VAS
Abbreviations: 95%CI, 95% confidence interval; HIV, Human Immunodeficiency Virus; P, P-value; Ref, Reference; y, year
